# Cyberbullying and Gambling Disorder: Associations with Emotion Regulation and Coping Strategies

**DOI:** 10.1007/s10899-022-10160-4

**Published:** 2022-10-01

**Authors:** Ana Estévez, Laura Macía, Hibai López-González, Janire Momeñe, Paula Jauregui, Nerea Etxaburu, Roser Granero, Fernando Fernández-Aranda, Gemma Mestre-Bach, Cristina Vintró-Alcaraz, Lucero Munguía, Isabel Baenas, Teresa Mena-Moreno, Bernat Mora-Maltas, Eduardo Valenciano-Mendoza, Susana Jiménez-Murcia

**Affiliations:** 1 Psychology Department, Faculty of Psychology and Education, University of Deusto, Apartado 1, 48080 Bilbao, Spain; 2 Departament of Library, Information Science, and Communication, University of Barcelona, Barcelona, Spain; 3 Departament de Psicobiologia I Metodologia, Universitat Autònoma de Barcelona, Barcelona, Spain; 4 Ciber Fisiopatologia Obesidad y Nutrición (CIBERobn), Instituto Salud Carlos III, Barcelona, Spain; 5 Psychoneurobiology of Eating and Addictive Behaviors Group, Institut d’Investigació Biomèdica de Bellvitge (IDIBELL), Barcelona, Spain; 6 Department of Psychiatry, Bellvitge University Hospital-IDIBELL and CIBERObn, C/Feixa Llarga S/N, 08907 Hospitalet de Llobregat, Barcelona, Spain; 7 Department of Clinical Sciences, School of Medicine and Health Sciences, University of Barcelona, Barcelona, Spain; 8 Universidad Internacional de La Rioja, La Rioja, Spain

**Keywords:** Gambling disorder, Cyberbullying, Emotion regulation, Coping strategies, Adolescence, Young

## Abstract

The presence of unsuitable coping and emotion regulation strategies in young populations with gambling disorder (GD) and in those who have experienced cyberbullying victimization has been suggested. However, this association has not been explored in depth. In this study, our aim was to analyze individual differences in emotion regulation, coping strategies, and substance abuse in a clinical sample of adolescents and young adult patients with GD (n = 31) and in a community sample (n = 250). Furthermore, we aimed to examine the association between cyberbullying and GD. Participants were evaluated using the Cyberbullying Questionnaire-Victimization, the Canadian Adolescent Gambling Inventory, the Coping Strategies Inventory, the Difficulties in Emotion Regulation Scale, the Alcohol Use Disorders Identification Test and the Drug Use Disorders Identification Test. Structural Equation Modeling was used to explore associations between these factors in a community sample and in a clinical group. In both groups, exposure to cyberbullying behaviors was positively associated with higher emotion dysregulation and the use of maladaptative coping styles. Our findings uphold that adolescents and young adults who were victims of cyberbullying show difficulties in emotion regulation and maladaptive coping strategies when trying to solve problems. The specific contribution of sex, age, gambling severity, emotion regulation, and coping strategies on cyberbullying severity is also discussed. Populations at vulnerable ages could potentially benefit from public prevention policies that target these risk factors.

**Supplementary Information:**

The online version contains supplementary material available at 10.1007/s10899-022-10160-4.

## Introduction

The prevalence of problematic gambling behaviors among adolescents and young adults is rising in many countries, bringing with it significant negative consequences in terms of social, familial, and occupational functioning (Calado et al., 2017a; Ferrara et al., 2018) Adolescents with problematic gambling have shown, among other aspects, deficits in concentration, increased distress, depressive symptoms, substance use, eating disorder psychopathology, and lower self-stem (Afifi et al., 2016; Marco & Tormo-Irun, 2018; Nigro et al., 2017; Shead et al., 2010).

Researchers have also posited that maladaptive emotion regulation strategies and dysfunctional coping skills are relevant risk factors in the development and maintenance of psychopathology and (Gross & Muñoz, 1995; Russell et al., 2012; Schreiber et al., 2012), specifically, in the case of adolescent problem gambling (Calado et al., 2017b; Estevez et al., 2014; Jauregui et al., 2016). Therefore, difficulties in controlling gambling behaviors may be influenced by deficits in coping skills, especially by using emotion-focused coping, such as escape or avoidance (Williams et al., 2012). Gambling may be used as a coping mechanism to escape from life difficulties and negative affect because, among other aspects, it provides immediate relief and distraction (Farrelly et al., 2007; Torres et al., 2013; Wood & Griffiths, 2007).

One of the difficulties that certain young people have to face is to experience a victimization process derived from bullying or its online modality, cyberbullying (Musharraf et al., 2018). Cyberbullying has been understood as “any behavior performed through electronic or digital media by individuals or groups that repeatedly communicates hostile or aggressive messages intended to inflict harm or discomfort on others”. (Tokunaga, 2010). Victims of cyberbullying are characterized by lower levels of emotional self-efficacy (Olenik-Shemesh & Heiman, 2014) and a more prevalent use of emotion-focused coping skills (focused on suppress or control negative emotions instead of change the stressor) and emotional suppression (Vranjes et al., 2018). Data suggest that being continually exposed to these kinds of traumatic or stressful life events may predispose individuals to develop disordered gambling (Brydges et al., 2015; Roberts et al., 2017), in addition to other psychopathological disorders, such as depression (Bottino et al., 2015). Regarding substance use, although some authors suggest the existence of an association between cyberbullying and substance use (Ybarra et al., 2007; Zsila et al., 2018), others have not found a link between these two behaviors (Selkie et al., 2015).

To date, the relationship between GD, being victim of cyberbullying, and the underlying psychological mechanisms that could be related to this association have been sparsely studied. As such, the aims of the present study were: (a) to estimate the prevalence of cyberbullying in both a sample of adolescents and young adults with GD and a community sample; (b) to analyze individual differences in emotion regulation, coping strategies, and substance abuse between a clinical sample of patients with GD and a community sample; and (c) to examine the underlying associations between cyberbullying and GD in both samples, considering direct and indirect (mediational) effects.

## Methods

### Sample and Procedure

The current study was conducted between December 2017 and April 2018. Both a clinical and community samples were recruited for this study. The clinical participants included *n* = 31 young patients who voluntarily asked for treatment at the outpatient specialized Gambling Disorder Unit at Bellvitge University Hospital in Barcelona, Spain. These patients were diagnosed according to DSM-5 criteria, by means of face-to-face interview (American Psychiatric Association, 2013). Most of the participants into this subsample were men (*n* = 28, 90.3%), and all were born in Spain. The mean for the chronological age was 20.8 years (SD = 2.4).

The community sample (*n* = 250) was recruited from secondary education schools from the Basque Country region in Spain following convenience sampling. Most of the participants into this subsample were born in Spain (*n* = 224, 89.6%), and distribution of sex was *n* = 126 men (49.6%) versus *n* = 124 women (%). The mean for the chronological age was 18.2 years (SD = 4.9). Invitations were sent out to local schools and a research team member travelled to participating centers to administer the paper-and-pencil questionnaires in person. Students completed the survey in their classrooms individually. The passing of the tests lasted approximately 40–50 min. During the completion of the protocols, members of the research team were present to resolve any doubts that might arise. The survey included general information regarding the study purposes. Minors had their consent forms signed by their parents/tutors prior to participating in the study.

Participation in this study had no compensation whatsoever for the people who participated. All schools received a general feedback report. This study was carried out in accordance with the latest version of the Declaration of Helsinki. The Ethics Committee of University of Deusto approved the study (ref number ETK-13/15-16), and signed informed consent was obtained from all participants.

### Measures

#### Cyberbullying Questionnaire-Victimization (CBQ-V) (Estévez et al., 2010)

This instrument contains 11 items that evaluate different forms of cyberbullying. It uses a Likert scale ranging from "0 = never" to "2 = often" (e.g., *writing embarrassing jokes, rumors, gossip, or comments about a classmate on the Internet*). This instrument was validated by confirmatory factorial analysis, in a one-factor structure, showing a good model fit. It has an adequate internal consistency index, with a Cronbach alpha of α = 0.95. In this study, the exposition to cyberbullying was considered absent for raw total score equals to 0 on the CBQ-V, while it was considered present using a raw total score higher than 0. Consistency in the sample of the study was adequate (α = 0.79).

#### Canadian Adolescent Gambling Inventory (CAGI) (Tremblay et al., 2010) Spanish Validation by Jiménez-Murcia et al. (2017) 

This is a self-report instrument designed for teenagers that measures the consequences derived from gambling behavior in two different sections. It includes 20 items measured on a 6-point scale to analyze the frequency, time and type of gambling, as well as the amount of money or valuables lost as a result of gambling (e.g., *do you prefer to hang out with friends who gamble/bet?*). It also includes 24 items measured on a four-point scale to measure the severity of one’s gambling problem, loss of control over gambling behavior, and the psychological, social and economic consequences derived from it (e.g., *how often have you borrowed money from family, friends, or others to gamble/bet?*). It also includes a subscale that measures gambling severity (GPSS) through nine of the items that make up the CAGI. It shows adequate psychometric indexes, with satisfactory reliability (internal consistency, Cronbach’s alpha, α  = 0.91), satisfactory convergent validity as measured by correlation with South Oaks Gambling Screen (r  = 0.74), and excellent classification accuracy (AUC  = 0.99; sensitivity = 0.98; and specificity = 0.99). Consistency in the sample of the study was excellent (α   = 0.96).

#### Coping Strategies Inventory (CSI) (Tobin et al., 1989) Spanish Validation by Cano et al. (2007)

The original scale was made up of 72 self-administered items. The Spanish adaptation reduced the original scale to 40, removing items that showed less factor loading, and an added measure of perceived self-efficacy in coping. The scale consists of eight 5-item subscales, with scores from 0 (not at all) to 4 (totally). The instrument has a hierarchical structure, composed of eight primary subscales, four secondary subscales, and two tertiary subscales. The eight subscales are: problem solving (e.g., *I stood my ground and fought for what I wanted*);, cognitive restructuring (e.g., *I told myself things that helped me feel better*), social support (e.g*., I found somebody who was a good listener*), emotional expression (e.g., *I let out my feelings to reduce the stress*), problem avoidance situation (e.g., *I went along as if nothing were happening*);, wishful thinking (e.g., *I hoped the problem would take care of itself*), social withdrawal (e.g., *I tried to keep my feelings to myself*), and self-blame (e.g., *I realized that I brought the problem on myself*). These subscales are further integrated into four additional secondary subscales: problem focused engagement, emotion focused engagement, problem focused disengagement, and emotion focused disengagement. Finally, it features two tertiary subscales: engagement and disengagement.

The CSI shows good psychometric properties, with Cronbach’s alphas ranging from 0.75 to 0.89 in the eight primary subscales in the Spanish validation. In the present study, consistency was between adequate (α = 0.75 for F1 social withdrawal) and very good (α = 0.90 for F3 adequate global) (Table 1 includes the Cronbach alpha for all the scales).

**Table 1:** Description of the study variables and comparison between groups defined for presence-absence of exposure to cyberbullying behaviors (separate descriptive data for community and clinical subsamples)

		Community sample				*p*	Clinical sample				*p*
		CyberB = no *n* = 154		CyberB = yes *n* = 96			CyberB no *n* = 21		CyberB = yes *n* = 10		
		*n*	*%*	*n*	*%*		*n*	*%*	*n*	*%*	
*Sex*											
Female		75	48.7%	49	51.0%	.719	2	9.5%	1	10.0%	.967
Male		79	51.3%	47	49.0%		19	90.5%	9	90.0%	
*Education level*											
Middle school		0	0%	0	0%	.906	4	19.0%	2	20.0%	
High school—9th grade		38	24.7%	21	21.9%		0	0%	0	0%	
High school—10th grade		23	14.9%	15	15.6%		0	0%	0	0%	
High school—11th grade		12	7.8%	6	6.3%		4	19.0%	1	10.0	
High school—12th grade		81	52.6%	54	56.3%		13	62.0%	7	70.0%	
*Origin*											
Spain		138	89.6%	86	89.6%	.995	17	81.0%	10	100%	.193
Immigrant		16	10.4%	10	10.4%		4	19.0%	0	0%	
		Mean	SD	Mean	SD	*p*	Mean	SD	Mean	SD	*p*
Age (years-old)		18.27	4.96	18.10	4.78	.791	21.14	2.56	20.10	1.91	.262
GPSS: Gambling problem severity	.964	1.19	2.97	0.99	2.23	.561	11.24	7.45	9.50	7.78	.554
DERS: Non-acceptance emotion	.870	10.05	4.50	12.51	5.06	.001*	15.86	5.54	15.60	6.82	.911
DERS: Difficulties directed beh	.793	11.93	4.39	13.67	4.44	**.003**	14.33	4.15	15.20	3.94	.585
DERS: Impulse control difficulties	.811	10.83	3.89	13.23	5.15	.001*	13.57	4.61	15.10	5.15	.413
DERS: Lack emotional awareness	.824	16.99	5.54	18.02	5.56	.153	18.38	5.18	19.70	4.99	.508
DERS: Limited emotion regulation	.854	14.34	5.35	17.35	6.60	.001*	19.62	7.30	18.50	6.49	.683
DERS: Lack of emotional clarity	.806	9.92	4.14	12.06	4.15	.001*	12.38	4.63	14.70	3.89	.182
DERS: Total score	.911	74.05	18.40	86.84	19.35	.001*	94.14	20.75	98.80	18.21	.549
CSI; F1st: problem solving	.831	12.47	4.98	12.29	4.12	.773	10.19	5.42	10.40	5.06	.919
CSI; F1st: cognitive restructure	.788	9.94	5.28	9.96	4.50	.971	10.52	4.02	9.20	4.66	.422
CSI; F1st: emotional expression	.818	8.94	4.88	9.61	5.03	.291	8.81	4.93	11.90	5.67	.130
CSI; F1st: social support	.821	12.12	5.50	11.74	4.93	.577	10.10	5.83	11.70	4.35	.447
CSI; F1st: problems avoidance	.751	7.42	4.44	8.23	4.67	.172	8.43	5.11	7.30	4.83	.564
CSI; F1st: cognitive desiderate	.844	11.32	5.77	13.34	4.80	.005*	14.33	5.86	14.10	6.31	.920
CSI; F1st: self-criticism	.841	6.49	4.87	8.29	4.53	.004*	13.05	6.05	12.80	6.03	.916
CSI; F1st: social withdrawal	.752	6.14	4.67	7.39	4.27	.035*	9.05	5.28	8.70	5.77	.869
CSI; F2nd: adequate problems	.870	22.44	9.27	22.23	7.71	.851	20.71	8.56	19.60	8.83	.740
CSI; F2nd: adequate emotions	.865	21.06	9.23	21.35	8.58	.805	18.90	10.16	23.60	9.66	.232
CSI; F2nd: non-adequate problems	.815	18.71	8.58	21.57	7.38	.007*	22.76	8.63	21.40	8.76	.686
CSI; F2nd: non-adequate emotions	.851	12.62	8.50	15.68	7.11	.004*	22.10	9.59	21.50	10.65	.877
CSI; F3rd: adequate, global	.908	43.52	16.89	43.60	14.59	.968	39.62	17.64	43.20	17.22	.599
CSI; F3rd: non-adequate, global	.878	31.26	14.56	37.25	12.00	.001*	44.86	17.39	42.90	18.80	.777
Alcohol: AUDIT total score	.854	3.91	4.53	5.99	6.06	.002*	3.76	3.65	3.50	1.18	.827
Other drugs: DUDIT total score	.929	3.21	6.68	3.98	7.72	.404	2.33	5.99	4.80	7.74	.337

*SD* standard deviation, *CyberB* exposure to cyberbullying behaviors, *F1st* first order factor, *F2nd* second order factor, *F3rd* third order factor

#### The Difficulties in Emotion Regulation Scale (DERS) (Gratz & Roemer, 2004) Spanish Validation by Hervás and Jódar (2008)

This instrument is made up of 36 items that gauge a number of factors concerning optimal emotion regulation (e.g., *when I’m upset, I become angry with myself for feeling that way*). Each item is evaluated on a 5-point Likert scale ranging from ‘Almost never’ (0–10% of the time) to Almost always (90–100% of the time). This scale comprises six latent factors: lack of emotional awareness, non-acceptance of emotional responses, lack of emotional clarity, difficulties engaging in goal-directed behavior, lack of emotional control, and impulse control difficulties. The previously reported psychometric properties of the instrument were adequate (Cronbach’s alpha of 0.93; range = 0.73–0.91, with a test–retest reliability of 0.88 in a 4–8-week period). Its six-factor structure has been validated in Spanish (Gómez-Simón et al., 2014). In the sample of the study, consistency was between adequate (α = 0.79 for “difficulties engaging in goal-directed behavior”) and excellent (α = 0.91 for total score) (Table 1 includes the Cronbach alpha for all the scales).

#### Alcohol Use Disorders Identification Test (AUDIT) (Saunders et al., 1993) Spanish Validation by Martínez (1999)

The AUDIT was developed as a simple screening method for excessive alcohol consumption. Internal consistency has been found to be high, and test–retest data have pointed to high reliability (0.86) and a sensitivity of around 0.90. Specificity in different settings and for different criteria averages 0.80 or more (Martínez, 1999). In this study, cutoff points of 8 and 20 were used to identify individuals with alcohol abuse and alcohol dependence, respectively (Martínez, 1999). Cronbach alpha in sample was very good (α = 0.85).

#### Drug Use Disorders Identification Test (DUDIT) (Berman et al., 2005)

This is an 11-item screening instrument developed to identify non-alcohol drug use patterns and various drug-related problems based on DSM-IV-TR criteria (American Psychiatric Association, 2000). The first nine items are scored on a 5-point Likert scale ranging from 0 to 4, and the last two are scored on 3-point scales (values of 0, 2, 4). Total scores can range from 0 to 44, with higher scores being indicative of more severe drug problems. It shows adequate reliability indices, with a Cronbach alpha of 0.93 in general population samples and 0.80 in clinical samples. Cronbach alpha in sample was excellent (α = 0.93).

#### Other Sociodemographic and Clinical Variables

Additional demographic, clinical, and social/family variables related to gambling were taken in the clinical group using a semi-structured face-to-face clinical interview described elsewhere (Jiménez-Murcia et al., 2007).

### Statistical Analysis

Statistical analysis was carried out with Stata16 for Windows. Firstly, categorical variables were compared between participants exposed to the presence of cyberbullying through chi-square tests (*χ*^2^), while quantitative variables were compared between both groups with t-test procedures.

Secondly, the association between cyberbullying severity (measured as the raw total score on the cyberbullying questionnaire) with the other clinical variables was estimated through partial correlations (R) adjusted for the covariates sex and age. For these estimations, and based on the strong association between significance and sample sizes, the relevance of the correlations was based on coefficient effect size (Rosnow & Rosenthal, 1996).

The specific contribution of the variables sex, age, gambling severity (GPSS-total), emotion regulation (DERS-total) and coping (CSI-adequate global and CSI-non adequate global) on cyberbullying severity was estimated through negative binomial regressions. These models constitute a type of generalized linear model in which the dependent variable is defined as a count of the number of times an event occurs, and it can be considered an extension of the Poisson regression for over-dispersed outcomes (Dupont, 2009). In this study, the negative binomial regressions were adjusted in two blocks/steps: (a) first block/step entered and set the variables sex, age, gambling severity, emotion regulation scores and coping strategy scores; and (b) second block/step added and tested the interaction parameters defined between the sex with the other clinical measures and between the age with the other clinical measures. After valuing the interaction parameters added to the second block/step, a final model was considered which retained only those significant interaction terms (*p* ≤ 0.05), interpreting the main effects for the non-significant interactions and the single effects for the significant interactions.

Finally, path-analysis explored the underlying mechanisms between age, coping strategies (CSI-adequate global and CSI-non adequate global) and emotion regulation (DERS-total) with gambling severity (GPSS-total) and cyberbullying severity (raw total score on the cyberbullying questionnaire). Path analysis constitutes an extension of multiple regression modeling, which can be used to estimate the magnitude and significance of hypothesized associations in a set of variables including mediational relationships (direct and indirect effects) (MacCallum & Austin, 2000). In this study, the path analysis was defined via structural equation modeling (SEM) using the maximum-likelihood method (MLE). Goodness-of-fit was tested with chi-square tests (χ^2^), the root mean square error of approximation (RMSEA), the Bentler’s Comparative Fit Index (CFI), the Tucker–Lewis Index (TLI), and the standardized root mean square residual (SRMR). (Bentler, 1990): *p* > 0.05 for χ^2^ test, RMSEA < 0.08, TLI > 0.9, CFI > 0.9 and SRMR < 0.1 was considered adequate fit. The global predictive capacity of the model was measured by the coefficient of determination (CD).

In this work, Holm’s method was also used to control Type-I error due to multiple statistical comparisons (this procedure is included in Family-wise error rate stepwise techniques and it has been demonstrated to be a more powerful test than Bonferroni correction) (Holm, 1979). In addition, due to the large set of variables, global measures were selected for the predictive models (negative binomial) and the path-analyses. Finally, all the analyses were stratified by the origin of the sample (community versus clinical), with the aim of assessing differences in patterns due to the origin of the sample and to allow for generalization to original populations.

## Results

### Characteristics of the Sample and Comparison Between Participants with and Without Cyberbullying

The number of patients who met positive screening score for cyberbullying in the community sample *n* = 96 (prevalence = 38.4%), compared to *n* = 10 into the clinical sample (prevalence = 32.3%) (no significant difference was found in the prevalence estimates between the groups: χ^2^ = 0.44, *df* = 1, *p* = 0.506). Comparing those who reported to be exposed to cyberbullying behaviors (*n* = 96 in the community sample versus *n* = 10 in the clinical sample) no differences emerged in the cyberbullying raw total scores: a mean equal to 1.9 (SD = 1.5) was found in the community sample versus 2.4 (SD = 2.3) in the clinical sample (T = 1.03, *df* = 104, *p* = 0.303).

Table 1 includes a description of the study variables stratified by the origin of the sample, and a comparison based on the presence/absence of exposure to cyberbullying behaviors (separate descriptive are reported also for community and clinical samples). No significant differences in sociodemographic variables were obtained comparing participants who reported exposure to cyberbullying, independent of the origin of the sample. In addition, in the clinical sample no differences between the groups were obtained in the clinical measures used. However, in the community sample, exposure to cyberbullying behaviors was related to higher emotion dysregulation, difficulties in coping strategies (higher scores in the first-order factors cognitive desiderate-self, criticism-social and withdrawal scales, in the second order factors non-adequate problems and emotions, and in the third order factor non-adequate driving), and higher scores in the alcohol use/abuse scale.

### Association Between Cyberbullying Severity and Clinical Measures

Table 2 includes the correlation matrix with the partial correlations (adjusted for sex and age) estimating the association between cyberbullying with gambling severity, emotion regulation, coping strategies and substance abuse. In the community sample, higher cyber-bulling severity was related to higher emotion dysregulation (concretely, in the DERS limited emotion regulation, lack of emotional clarity and total scale scores). In the clinical sample, higher cyber-bulling severity was related to lower gambling severity, higher level of lack of emotional clarity and most dysfunctional coping strategies (concretely, in the first order factors problem solving, emotional expression, social support and social withdrawn, in the second order factor adequate problems and emotions, and in the third order factor adequate driving global).

**Table 2:** Associations between cyberbullying severity and gambling severity, emotion regulation, coping strategies and substance abuse: partial correlations adjusted for sex and age

	Community (*n* = 250)	Clinical (*n* = 31)
GPSS: gambling severity	− .018	− **.324**^**†**^
DERS: non-acceptance emotion	.210	− .017
DERS: difficulties directed behavior	.149	− .062
DERS: impulse control difficult	.229	.090
DERS: lack of emotional awareness	.066	.137
DERS: limited emotion regulation	**.274**^**†**^	− .010
DERS: lack of emotional clarity	**.273**^**†**^	**.283**^**†**^
DERS: total score	**.300**^**†**^	.101
CSI; F1st: problem solving	− .035	**.251**^**†**^
CSI; F1st: cognitive restructure	− .042	.189
CSI; F1st: emotional expression	.018	**.303**^**†**^
CSI; F1st: social support	− .022	**.258**^**†**^
CSI; F1st: problems avoidance	.095	.087
CSI; F1st: cognitive desiderate	.074	.107
CSI; F1st: self-criticism	.183	.084
CSI; F1st: social withdrawal	.134	**.240**^**†**^
CSI; F2nd: adequate problems	− .045	**.247**^**†**^
CSI; F2nd: adequate emotions	− .003	**.295**^**†**^
CSI; F2nd: non-adequate problems	.103	.122
CSI; F2nd: non-adequate emotions	.183	.182
CSI; F3rd: adequate global	− .026	**.293**^**†**^
CSI; F3rd: non-adequate global	.169	.161
Alcohol: AUDIT total score	.171	− .155
Other drugs: DUDIT total score	.091	.007

*F1st* first order factor, *F2nd* second order factor, *F3rd* third order factor

^†^Bold: effect size in the medium-mean (|*R*|> 0.24) to high-large (|*R*|> 0.37) range

Table 3 includes the negative binomial regressions exploring the specific contribution of sex, age, gambling severity, emotion regulation and coping strategies (defined as independent models) on cyberbullying severity (dependent variable). In the community sample, the first model showed no significant interaction parameter between sex and age with the remaining clinical variables. The main effects of the final model showed that the only significant contributor was the DERS-total score (B = 0.03, *p* < 0.001). In the clinical sample, the first model showed that the interaction parameter between age with gambling severity obtained significance (*p* = 0.029), indicating that the association between gambling and cyberbullying severity differed depending on the patients’ age. The single effects obtained in the final model were interpreted, which indicated the association between the gambling severity with the cyberbullying level was higher for the older patients: a) among young age patients (age defined as the percentile 5 in the sample), no association emerged between gambling level with cyberbullying; among middle age patients (percentile 50 for age) and among old age patients (percentile 95 for age), the intensity of the relationship increased from B =  − 0.22 (*p* = 0.009) to B =  − 0.56 (*p* = 0.012).

**Table 3:** Predictive capacity of sex, age, gambling severity, emotion regulation and coping on cyberbullying severity: negative binomial regression

	*B*	*SE*	95%CI (*B*)		*Wald*	*P*
*Community subsample (n* = 250*)*						
First model: exploring interaction parameters						
Sex (0 = female; 1 = male)	0.020	0.245	− 0.459	0.499	0.007	.934
Age (years-old)	0.018	0.026	− 0.033	0.068	0.462	.497
Gambling severity (CAGI-GPSS total)	0.155	0.473	− 0.771	1.081	0.108	.743
DERS: Total score	0.028	0.007	0.015	0.042	16.629	< .001*
CSI; F3rd: adequate global	− 0.002	0.008	− 0.019	0.015	0.056	.812
CSI; F3rd: non-adequate global	0.014	0.010	− 0.007	0.035	1.778	.182
Interaction: gambling severity*Sex	0.189	0.250	− 0.300	0.679	0.575	.448
Interaction: gambling severity*Age	0.004	0.009	− 0.013	0.021	0.191	.662
Interaction: gambling severity*DERS-total	− 0.003	0.003	− 0.009	0.003	0.810	.368
Interaction: gambling severity*CSI-F3rd_adequate	0.001	0.004	-0.006	0.009	0.098	.754
Interaction: gambling severity*CSI-F3rd_non-adequate	− 0.007	0.005	− 0.017	0.002	2.167	.141
Final model						
Sex (0 = female; 1 = male)	0.031	0.232	− 0.423	0.486	0.018	.893
Age (years-old)	0.027	0.023	− 0.018	0.072	1.363	.243
Gambling severity (CAGI-GPSS total)	− 0.026	0.044	− 0.112	0.059	0.369	.543
DERS: Total score	0.027	0.006	0.014	0.039	17.420	< .001*
CSI; F3rd: adequate global	− 0.002	0.008	− 0.017	0.014	0.053	.818
CSI; F3rd: non-adequate global	0.009	0.010	− 0.010	0.028	0.927	.336
*Clinical subsample (n* = *31)*						
First model: exploring interaction parameters						
Sex (0 = female; 1 = male)	− 0.408	1.982	− 4.293	3.477	0.042	.837
Age (years-old)	0.703	0.414	− 0.109	1.515	2.877	.090
Gambling severity (CAGI-GPSS total)	2.162	1.079	0.046	4.277	4.012	.045*
DERS: Total score	0.010	0.058	− 0.104	0.124	0.029	.864
CSI; F3rd: adequate global	0.039	0.045	− 0.049	0.128	0.770	.380
CSI; Factor 3r: non-adequate global	0.045	0.045	− 0.043	0.134	1.005	.316
Interaction: gambling severity*Sex	0.084	0.159	− 0.230	0.395	0.525	0.600
Interaction: gambling severity*age	− 0.104	0.051	− 0.204	− 0.003	4.075	.044*
Interaction: gambling severity*DERS-total	0.001	0.004	− 0.008	0.009	0.041	.840
Interaction: gambling severity*CSI-F3rd_adequate	− 0.003	0.004	− 0.011	0.004	0.851	.356
Interaction: gambling severity*CSI-F3rd_non-adequate	− 0.003	0.005	− 0.012	0.006	0.455	.500
Final model						
Sex (0 = female; 1 = male)	− 0.829	1.591	− 3.948	2.291	0.271	.603
Age (years-old)	0.768	0.401	− 0.018	1.554	3.670	.055
Gambling severity^a^						
Young age (P_05_ = 17 years-old)	0.224	0.155	− 0.080	0.528	2.084	.149
Middle age(P_50_ = 21 years-old)	− 0.225	0.087	− 0.395	− 0.055	6.752	.009*
Old age (P_95_ = 24 years-old)	− 0.562	0.225	− 1.002	− 0.122	6.261	.012*
DERS: Total score	0.017	0.023	− 0.029	0.063	0.544	.461
CSI; F3rd: adequate global	0.010	0.032	− 0.053	0.073	0.090	.764
CSI; F3rd: non-adequate global	0.030	0.034	− 0.037	0.098	0.764	.382
Interaction: gambling severity*age	− 0.112	0.051	− 0.213	− 0.012	4.782	.029*

^*^Bold: significant parameter (.05 level). F3rd: third order factor

^a^Single effects for gambling severity for the percentiles of age 5, 50 and 95 in the group

### Pathways Analysis

**Fig. 1:**
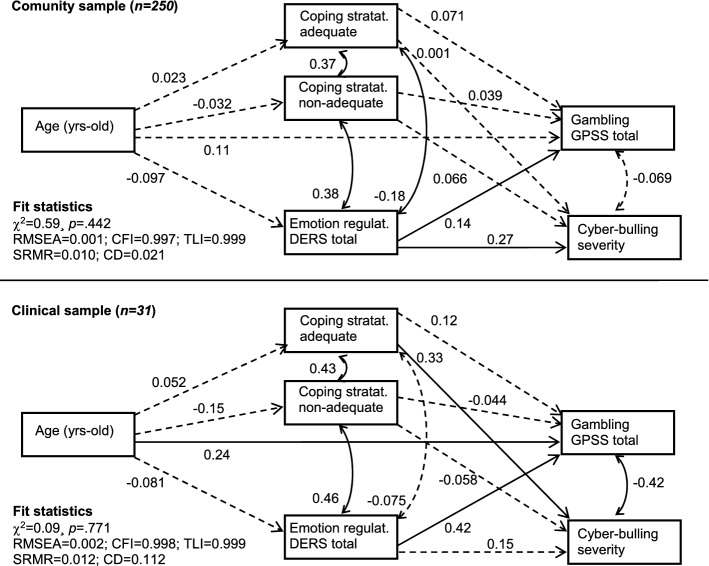
Path diagrams with the standardized coefficients obtained in the SEM. *Note.* Continuous line: significant parameter (.05 level). Dash-line: non-significant parameter

Figure 1 includes the path-diagrams for the SEM. Adequate goodness-of-fit was obtained in the two models adjusted for the community and clinical samples. Table S1 includes the complete results of the models, including the tests for direct, indirect and total effects.

In the community sample, cyberbullying severity was directly related to the DERS-total score (worse emotion regulation predicted higher cyberbullying). In the clinical sample, cyberbullying severity was directly related to the adequate-driving coping strategies, while an indirect effect was also obtained between age and gambling severity with cyberbullying (older age predicted higher gambling severity, while higher gambling severity was related to lower cyberbullying severity).

## Discussion

Despite the positive benefits of internet and social media among young age people (including quick access to information, access to teaching and learning resources, and increased levels of social support), the frequency of bullying online has become a widespread common problem for youths around the world, resulting in a high prevalence of young people affected by cybervictimization. This bullying is quite different from “traditional” forms, in the sense that it is not confined to school or the playground and there is no escape for victims (young people connect usually through social media and simply go offline is not an option for many individuals). Moreover, although cyberbullying is socially considered alarming with real severe consequences (including negative psychological health outcomes), not enough research has yet been conducted regarding its correlates. This study estimates the presence of cyberbullying behavior in a clinical sample of young patients who met criteria for GD and in a community sample, as well as the potential association of this form of bullying with emotion regulation profile, coping strategies, substance use-abuse and problem gambling severity. The main results of the study showed a high prevalence of individuals who reported presence of cyberbullying behavior in both samples (around 1 in 3 individuals in our work have experienced cyberthreats online), and that the underlying mechanism and correlates of this victimization was related to the origin of the samples (clinical versus population-based).

It is well known that emotions play an essential role in the way how people manage their behavior and social interactions (Cole et al., 2004). In our community sample, exposure to cyberbullying behaviors was positively related to higher emotion dysregulation. In this regard, higher scores in cyberbullying were associated with higher levels of lack of emotional clarity in the clinical sample. This result dovetails with previous studies also reporting a link between these two factors (Hemphill & Heerde, 2014; Hemphill et al., 2015; Vranjes et al., 2018). Emotion dysregulation, specifically, has been described to make a relevant contribution differentiating bullies and victims from individuals not exposed to these problems (Shields & Cicchetti, 2001). Therefore, difficulties in the management of emotions in social interactions could be considered as a predictor of cyberbullying (Baroncelli & Ciucci, 2014).

In both the clinical and community samples, higher cyberbullying severity was also related to the use of maladaptative coping styles. Previous data have suggested that coping strategies in young people may be relevant in the association between bully victimization and their psychological well-being (Garnefski & Kraaij, 2014). Data has identified the feeling of ineffectiveness in solving problems in bully victims, as well as passive, emotionally-oriented and avoidant coping styles (Hansen et al., 2012; Tenenbaum et al., 2011). Specifically, coping strategies more associated with bullying were rumination and catastrophizing (Garnefski & Kraaij, 2014).

Regarding the SEM, in the community sample, worse emotion regulation predicted higher cyberbullying, while in the clinical sample, cyberbullying severity was directly related to a lack of adequate-driving coping strategies. These findings would again demonstrate that both emotional regulation and coping strategies are closely associated with cyberbullying (Ittel et al., 2014).

The findings of this study also present an association, in the community sample, between alcohol use/abuse and having experienced cyberbullying. This observation coincides with other research highlighting an association between victimization through bullying and high-risk behaviors, such as alcohol and substance use (Khantzian, 1997; Maniglio, 2009).

In agreement with the risk taking patterns presented by those subjects who have experienced bullying (Poon, 2016), we expected to find higher levels of severity and higher difficulties in emotion regulation and coping processes in the group with a diagnosis of GD. Studies in this line suggest that victims of bullying show a tendency to overestimate benefits, to underestimate risks and to present higher impulsivity levels, common features also found in GD (Poon, 2016; Steward et al., 2017) . However, both the control group and the clinical group had no differences in GD severity when comparing those subjects who had undergone cyberbullying and those who had not. One explanation could be that these populations opt for other types of behavior, such as alcohol consumption, as mentioned above (Khantzian, 1997; Maniglio, 2009).

## Clinical Implications

Cyberbullying represents a high prevalent dangerous form of victimization characterized by harassment and humiliation that can be experienced through various mediums (e.g., e-mail, chats, mobile phones), with harmful correlates in maladaptative coping strategies and emotion dysregulation. Experiencing cyberbullying could have a long-lasting impact on the victims, who would be predisposed to wider mental health problems such as low self-stem, somatic symptoms, aggression, depression, anxiety and substance/behavioral related disorders. The findings of the present study highlight the importance of the exposure to cyberbullying in the emotion regulation and the use of adaptative coping strategies during adolescence and young adulthood in population-based samples and in a clinical sample of patients who met criteria for GD, The empirical evidences regarding the impact of this bullying form on the variables analyzed in the work allows developing accurate, reliable, and valid assessment instruments and planning useful preventive and therapeutic interventions high-risk individuals exposed to this type of victimization (in both clinical and community settings). Therefore, as other authors have suggested, one of the aims of bullying and cyberbullying prevention plans should be to promote emotional and coping skills in the classroom (Garaigordobil & Martínez-Valderrey, 2018; Marikutty & Joseph, 2016; Schokman et al., 2014).

## Limitations and Strengths

There are limitations that should be considered when interpreting the results. First, the cross-sectional design of the present study does not allow for establishing conclusions regarding causality and the direction of the effects. Longitudinal studies are needed to give essential insights on the underlying mechanisms between these factors and the evolution and long-term effect of the associations under study. Second, this study has focused exclusively on the victims of cyberbullying, without exploring the clinical profile of the aggressors. In addition, it has not considered the time elapsed between the victimization process and the collection of the study data. Low sample size was also a methodological limitation (particularly for the clinical subsample) associated with reduced statistical power and increased likelihood of Type-II errors (results of this study should be interpreted in a more descriptive than inferential way, pending that future research with larger clinical samples confirm/validate our findings). Finally, emotion regulation and copying strategies were assessed using exclusively self-report measures, which are unable to fully capture the complexity of these constructs.

Regarding strengths, this study included an extensive assessment including standardized measures of the cyberbullying presence, the gambling severity, coping strategies and (dys)regulation emotion. Moreover, the statistical analysis of data recruited from GD patients and from a community sample allows to obtain evidences of the frequency of the cyberbullying victimization and the associations between the variables under study possible to generalize across clinical and also population-based settings.

## Supplementary Information

Below is the link to the electronic supplementary material.

Supplementary file1 (DOCX 16 kb)

## Data Availability

The datasets generated during and/or analysed during the current study are not publicly available due to confidentially reasons.
