# Supplementary material for: Cyberbullying and Gambling Disorder: Associations with Emotion Regulation and Coping Strategies
Source: J Gambl Stud. 2022 Oct 1;39(3):1399–416. doi: 10.1007/s10899-022-10160-4 (PMC10397144; doi:10.1007/s10899-022-10160-4)
Supplement: Supplementary file 1 — Supplementary file1 (DOCX 16 kb) [file 10899_2022_10160_MOESM1_ESM.docx]

*Table S1 (supplementary material) Direct, indirect and total effects in the SEM*

|  |  | Community sample | | | | | Clinical sample | | | | |
| --- | --- | --- | --- | --- | --- | --- | --- | --- | --- | --- | --- |
| Direct effects |  | *B* | *SE(B)* | *Z* | *p* | *St.(B)* | *B* | *SE(B)* | *Z* | *p* | *St.(B)* |
| Cop. non-adeq. | Age | 0.0752 | 0.2075 | 0.36 | .717 | 0.0229 | 0.3784 | 1.3002 | 0.29 | .771 | 0.0522 |
| GD severity | Cop. adequate | 0.0120 | 0.0122 | 0.98 | .325 | 0.0709 | 0.0522 | 0.0821 | 0.64 | .525 | 0.1203 |
|  | Cop. non-adeq. | 0.0075 | 0.0149 | 0.5 | .614 | 0.0386 | -0.0188 | 0.0914 | -0.21 | .837 | -0.0439 |
|  | Dysreg.Emot. | 0.0190 | 0.0099 | 1.98 | .047 | 0.1383 | 0.1576 | 0.0729 | 2.16 | .031 | 0.4154 |
|  | Age | 0.0594 | 0.0345 | 1.72 | .085 | 0.1072 | 0.7465 | 0.4666 | 1.6 | .11 | 0.2373 |
| CyberB | Cop. adequate | 0.0000 | 0.0056 | 0 | .996 | 0.0003 | 0.0327 | 0.0197 | 1.97 | .048 | 0.3310 |
|  | Cop. non-adeq. | 0.0061 | 0.0068 | 0.88 | .376 | 0.0658 | -0.0056 | 0.0218 | -0.26 | .797 | -0.0577 |
|  | Dysreg.Emot. | 0.0176 | 0.0046 | 3.87 | 0 | 0.2714 | 0.0126 | 0.0176 | 0.72 | .474 | 0.1461 |
| Cop. non-adeq. | Age | -0.0899 | 0.1803 | -0.5 | .618 | -0.0315 |  |  |  |  |  |
| Dysreg.Emot. | Age | -0.3911 | 0.2546 | -1.54 | .125 | -0.0967 | -0.6681 | 1.4841 | -0.45 | .653 | -0.0806 |
| Indirect effects |  | *B* | *SE(B)* | *Z* | *p* | *St.(B)* | *B* | *SE(B)* | *Z* | *p* | *St.(B)* |
| GD severity | Age | -0.0072 | 0.0068 | -1.06 | .287 | -0.0130 |  |  |  |  |  |
| CyberB | Age | -0.0074 | 0.0052 | -1.42 | .155 | -0.0283 | 0.0100 | 0.0475 | 0.21 | .834 | 0.0139 |
| Total effects |  | *B* | *SE(B)* | *Z* | *p* | *St.(B)* | *B* | *SE(B)* | *Z* | *p* | *St.(B)* |
| Cop. adequate | Age | 0.0752 | 0.2075 | 0.36 | .717 | 0.0229 | 0.3784 | 1.3002 | 0.29 | .771 | 0.0522 |
| GD severity | Cop. adequate | 0.0120 | 0.0122 | 0.98 | .325 | 0.0709 | 0.0522 | 0.0821 | 0.64 | .525 | 0.1203 |
|  | Cop. non-adeq. | 0.0075 | 0.0149 | 0.5 | .614 | 0.0386 | -0.0188 | 0.0914 | -0.21 | .837 | -0.0439 |
|  | Dysreg.Emot. | 0.0190 | 0.0099 | 1.98 | .047 | 0.1383 | 0.1576 | 0.0729 | 2.16 | .031 | 0.4154 |
|  | Age | 0.0522 | 0.0349 | 1.5 | .134 | 0.0942 | 0.6812 | 0.5118 | 1.33 | .183 | 0.2166 |
| CyberB | Cop. adequate | 0.0000 | 0.0056 | 0 | .996 | 0.0003 | 0.0327 | 0.0197 | 1.97 | .048 | 0.3310 |
|  | Cop. non-adeq. | 0.0061 | 0.0068 | 0.88 | .376 | 0.0658 | -0.0056 | 0.0218 | -0.26 | .797 | -0.0577 |
|  | Dysreg.Emot. | 0.0176 | 0.0046 | 3.87 | 0 | 0.2714 | 0.0126 | 0.0176 | 0.72 | .474 | 0.1461 |
|  | Age | -0.0074 | 0.0052 | -1.42 | .155 | -0.0283 | 0.0100 | 0.0475 | 0.21 | .834 | 0.0139 |
| Cop. non-adeq. | Age | -0.0899 | 0.1803 | -0.5 | .618 | -0.0315 | -1.0750 | 1.3078 | -0.82 | .411 | -0.1460 |
| Dysreg.Emot. | Age | -0.3911 | 0.2546 | -1.54 | .125 | -0.0967 | -0.6681 | 1.4841 | -0.45 | .653 | -0.0806 |

*Note.* B: un-standardized coefficient. SE: standard error. St.(B): standardized coefficient.
